# Aflatoxin-Induced *TP53 R249S* Mutation in HepatoCellular Carcinoma in Thailand: Association with Tumors Developing in the Absence of Liver Cirrhosis

**DOI:** 10.1371/journal.pone.0037707

**Published:** 2012-06-04

**Authors:** Stephanie Villar, Sandra Ortiz-Cuaran, Behnoush Abedi-Ardekani, Doriane Gouas, Andre Nogueira da Costa, Amelie Plymoth, Thiravud Khuhaprema, Anant Kalalak, Suleeporn Sangrajrang, Marlin D. Friesen, John D. Groopman, Pierre Hainaut

**Affiliations:** 1 International Agency for Research on Cancer, Lyon, France; 2 Karolinska Institute, Stockholm, Sweden; 3 National Cancer Institute, Bangkok, Thailand; 4 Bloomberg School of Public Health, Johns Hopkins University, Baltimore, Maryland, United States of America; 5 International Prevention Research Institute, Lyon, France; Drexel University College of Medicine, United States of America

## Abstract

Primary Liver Cancer (PLC) is the leading cause of death by cancer among males in Thailand and the 3^rd^ among females. Most cases are hepatocellular carcinoma (HCC) but cholangiocarcinomas represent between 4 and 80% of liver cancers depending upon geographic area. Most HCC are associated with chronic infection by Hepatitis B Virus while a G→T mutation at codon 249 of the *TP53* gene, *R249S*, specific for exposure to aflatoxin, is detected in tumors for up to 30% of cases. We have used Short Oligonucleotide Mass Analysis (SOMA) to quantify free circulating *R249S-*mutated DNA in plasma using blood specimens collected in a hospital case:control study. Plasma *R249S-*mutated DNA was detectable at low concentrations (≥67 copies/mL) in 53 to 64% of patients with primary liver cancer or chronic liver disease and in 19% of controls. 44% of patients with HCC and no evidence of cirrhosis had plasma concentrations of *R249S-*mutated DNA ≥150 copies/mL, compared to 21% in patients with both HCC and cirrhosis, 22% in patients with cholangiocarcinoma, 12% in patients with non-cancer chronic liver disease and 3% of subjects in the reference group. Thus, plasma concentrations of *R249S-*mutated DNA ≥150 copies/mL tended to be more common in patients with HCC developing without pre-existing cirrhosis (p = 0.027). Overall, these results support the preferential occurrence of *R249S-*mutated DNA in HCC developing in the absence of cirrhosis in a context of HBV chronic infection.

## Introduction

Liver cancer accounts for approximately 6% of all new cancer cases diagnosed worldwide; it is the fifth most common cancer in men and the eighth most common in females worldwide [1], [2]. The majority of primary liver cancers in adults are hepatocellular carcinoma (HCC; 90%) whereas cholangiocarcinomas (CC) account for about 9% of the cases, with peaks in incidence in parts of South-East Asia. Approximately 80% of HCCs and the resulting deaths occur in low and middle income countries of Eastern Asia, Sub-Saharan Africa and South America, where high incidences of Hepatitis B Virus (HBV) and/or Hepatitis C Virus (HCV) are endemic and where aflatoxin-contaminated staple foods are consumed [3]. CC develops in intra-hepatic billiary epithelial cells and is caused by the chronic infection of billiary ducts by liver flukes *Opisthorchis viverrini* and *Clornorchis Sinesis*. These liver flukes are endemic mainly in Eastern Asia, notably in Thailand, Laos, Vietnam and South-Eastern China [4]. According to a recent evaluation of the carcinogenic risk to humans by the International Agency for Research on Cancer Monographs, there is only limited evidence for association chronic HBV/HCV with risk of CC and no evidence for association with aflatoxin exposure [5], [6].

In Thailand, liver cancer is the leading cause of death from cancer among men (age-standardized incidence rates, world standard, ASR = 35.1) and the third leading cause in women (ASR = 16.6) [1]. The geographical distribution of cholangiocarcinoma matches the prevalence of chronic infection by *O. viverrini,* which is endemic in the north-east and rare in southern Thailand. In males, the age-standardized incidence rates (ASR, world standard) for HCC and CC, respectively, are 11.6 and 7.2 for Chiang Mai, 11.0 and 10.4 for Lampang, 12.5 and 67.5 for Khon Kaen, 8.3 and 2.2 for Bangkok and 5.9 and 0.4 for Songkhla [7]. In Thailand, nationwide infant vaccination against HBV began in 1992 [8], [9].

Aflatoxin exposure is a major risk factor for HCC in particular in regions where exposure to HBV is endemic. Aflatoxins are fungal toxins produced by *Aspergillus flavus* and *Aspergillus parasiticus*, which contaminate staple foods including groundnuts. Storage of crops in hot humid conditions can promote growth of the aflatoxin-producing fungi and results in increased accumulation of the toxin [10], [11]. Aflatoxin B_1_ (AFB_1_), the most abundant form, is metabolized by P450 enzymes in the liver to generate an epoxide which is highly reactive with DNA, forming adducts at N7 position of Guanine. Lack of repair of this lesion may lead to permanent DNA mutations, preferentially G to T transversions. A hotspot for mutation by AFB_1_ has been identified at codon 249 in the *TP53* suppressor gene (AGG to AGT, Arginine to Serine, *R249S*). Recent evidence confirms that this position is a preferential site for AFB_1_ adduct formation [12]. There is a remarkable ecological concordance between AFB_1_ exposure, chronic HBV infection and presence of *R249S* in HCC [13], [14]. However, how the mutant protein p.R249S contributes to hepatocarcinogenesis and cooperates with HBV in this process is still a matter of debate.

In Thailand, the main dietary sources of exposure to AFB_1_ are maize and groundnuts (peanuts). Individual exposure to AFB_1_ has been estimated to vary between 53 and 73 ng/kg/day, although this figure is likely to differ widely among geographic areas and ecological zones [11]. A recent estimate of the risk of HCC attributable to aflatoxin for Thailand has provided figures of 0.53–0.73 and 15.9–21.9/10^5^ person years, in HBsAg-negative and positive subjects, respectively [11]. In a recent study on a small group of surgically resected HCC patients at the National Cancer Institute, Bangkok, we reported *R249S* mutation in 7/26 (27%) cases, suggesting that the contribution of AFB_1_ to the burden of HCC in Thailand is far from negligible [15]. However, an earlier epidemiological study in Thailand using a albumin-adduct biomarker to assess aflatoxin exposure, failed to identify an aflatoxin-associated risk for HCC [16].

In previous studies, we and others have shown that circulating free DNA (CFDNA) from plasma is a suitable surrogate source of liver-derived DNA for detection of *R249S* mutations. Overall levels of CFDNA are higher in patients with liver cancer than controls (for review see [17]). In the Gambia and in the Qidong area of People’s Republic of China, two regions of high HBV prevalence and widespread AFB_1_ exposure, high plasma concentrations of *R249S*-mutated DNA were found to be strongly associated with HCC [18], [19]. In the Gambia, the use of a highly sensitive mass spectrometric technique (Short Oligonucleotide Mass Analysis, SOMA) has allowed detection of low levels *R249S*-mutated DNA in the plasma of asymptomatic HBV carriers, suggested to occur as a result of ongoing AFB_1_ exposure [20]. In Thailand, a previous study comparing HCC cases and controls has reported the SOMA detection of *R249S*-mutated DNA in 9/34 HCC cases (26%) and in 10/68 (14.7%) controls [21]. In this study, we have used plasma collected in a case-referent design at National Cancer Institute, Bangkok, to evaluate the association of *R249S*-mutated DNA plasma concentrations with the occurrence of HCC, CC or chronic liver disease. Our results suggest a preferential association between *R249S* and HCC developing in the absence of documented previous history of liver cirrhosis.

## Methods

### Ethics Statement

Written consent was obtained from all participants in the Thailand case-referent study and this study was approved by the Institutional Review Boards of the Thailand National Cancer Institute and the International Agency for Research on Cancer.

### Study Participants

This study has been carried out using protocols, specimens and data from the International Liver Cancer Study (ILCS), an international initiative that aims to contribute to intervention, prevention, early diagnosis and control of liver cancer through the understanding of its causes and mechanisms in different populations. ILCS Thailand is a case – referent study in which subjects were recruited from fifty five provinces all around the country from April 2008 to December 2009. Diagnosis of hepatocellular carcinoma and cholangiocarcinoma was based on concordant clinical examination and abdominal imaging. Individuals assigned to the reference group presented no clinical evidence of liver disease and were selected among the group of patients that came to the Institute for their annual check-up.

### Quantitation of *TP53 R249S* Mutation in CFDNA

Circulating free DNA was extracted from 1 mL of plasma using QiAmp circulating nucleic acid kit (Qiagen, Hilden, Germany) according to the manufacturer’s protocol for purification of Circulating DNA from 1 mL, 2 mL or 3 mL serum or plasma. Purified DNA was eluted from the QiAmp Silica column with water (2×50 µL) (PCR-grade, Sigma, St Louis, MO, USA). Quantitation of extracted DNA was performed by fluorimetry using picogreen (Molecular Probes, Eugene, OR, USA). *R249S*-mutated DNA was detected and quantified in reference to an internal standard plasmid by SOMA as described previously [20], [22]. Plasma concentrations were expressed as copies of *R249S*-mutated DNA per mL of plasma. The limit of determination for the method was 67 copies of *R249S*-mutated DNA/mL plasma.

### Statistical Analyses

Odds ratios (ORs), χ^2^ and p-values were calculated using STATA 11.1 (College Station, Texas). Odds ratios were adjusted for sex and age as well as for HBsAg and HCV-Ab.

## Results

### Characteristics of Study Population

**Figure 1:**
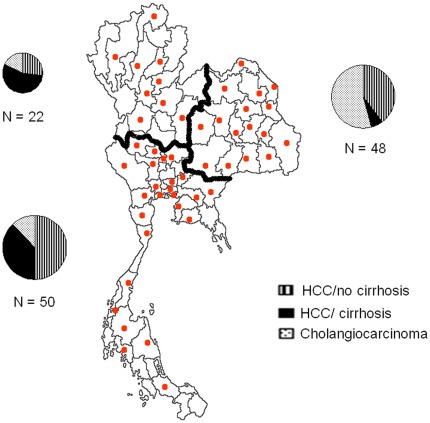
Geographic distribution of liver cancer cases. Dots represent the distribution by province. Pie charts describe the distribution of HCC/no cirrhosis (lines), HCC/cirrhosis (full black) and CC (small dots) among the Northwest, Northeast and Central-south parts of the country.

Demographic information and hepatitis infection status of cases of HCC without cirrhosis (HCC/no cirrhosis; n = 50), HCC with cirrhosis (HCC/cirrhosis; n = 34), cholangiocarcinoma (CC; n = 36), chronic liver disease (CLD; n = 56) and of the reference group (R; n = 133) are presented in Table 1. Median age was 55 years (range 42–74) for HCC/cirrhosis, 52 (33–71) for HCC/no cirrhosis, 57 (37–72) for CC and 53 (35–72) for CLD. Of note, 38% (19/50) of HCC/no cirrhosis were under 50 years of age, compared to 18% for HCC/cirrhosis. (p = 0.045). Male to female ratio was 4∶1 for HCC/no cirrhosis, 3∶1 for HCC/cirrhosis, 2∶1 for CC and 1.5∶1 for CLD. Figure 1 shows the geographic distribution of the place of residence of the cases at the time of diagnosis. Patients originated from all around the country, but were mainly from the North-East and the Central-South regions. HCC/cirrhosis cases tended to cluster in the Central-South region (mainly larger Bangkok area), as compared to HCC/no cirrhosis (predominantly rural) and to CC (predominantly originating from north-eastern Thailand).

**Table 1:** Characteristics of the study participants.

			Status n (%)				
			Hepatocellular carcinomawithout cirrhosis	Hepatocellular carcinomawith cirrhosis	Cholangicarginoma	Chronic liver disease	Reference group
**N**			**50**	**34**	**36**	**56**	**133**
	<40		5 (10)	0 (0)	1 (2.8)	5 (8.9)	5 (3.8)
**Age, years**	40–49		14 (28)	6 (17.6)	6 (16.7)	12 (21.4)	28 (21)
	50–59		18 (36)	19 (55.9)	13 (36.1)	26 (46.5)	51 (38.4)
	≥60		13 (26)	9 (26.5)	16 (44.4)	13 (23.2)	49 (36.8)
**Sex**	Men		41 (82)	26 (76.5)	23 (63.9)	34 (60.7)	77 (57.9)
	Women		9 (18)	8 (23.5)	13 (36.1)	22 (39.3)	56 (42.1)
	Negative		19 (38)	16 (47)	34 (94.4)	27 (48.2)	112 (84.2)
**HBsAg**	Positive		31 (62)	17 (50)	1 (2.8)	29 (51.8)	19 (14.3)
	N/A		0 (0)	1 (3)	1 (2.8)	0 (0)	2 (1.5)
	**OR [95% CI]** §		11.67 [4.99–21.3]	12.32 [4.43–34.27]	0.18 [0.02–1.04]	9.64 [4.31–21.57]	1.00
**Hepatitis C**	Negative		43 (86)*	23 (67.7)	34 (94.4)	43 (76.8)	133 (100)
	Positive		7 (14)*	11 (32.3)	2 (5.6)	13 (23.2)	0 (0)

§ OR for HBsAg positivity calculated in relation to the R group. * X_2_ test when compared HCC/no cirrhosis Vs HCC/cirrhosis (p value = 0.044).

HBV carriage, as assessed by detection of HBsAg, was significantly more frequent in HCC/no cirrhosis (62%; OR: 11.7, 95%CI [5.0–21.3]); HCC/cirrhosis (50%; OR: 12.3, 95%CI [4.4–34.3]) and CLD (52%; OR: 9.6, 95%CI [4.3–21.6]) as compared to R (14%; Reference; *p = *<0.001). Of note, the proportion of HBsAg-positive subjects in the R group was higher than expected based on current WHO health statistics for the Thai population [9]. Conversely, CC were less associated with HBsAg positivity than the R group (2.8%; OR: 0.2, 95%CI [0.02–1.04]). HCV seropositivity was confirmed in 14% of HCC/no cirrhosis and in 32.3% of HCC/cirrhosis (X_2_, *p = *0.044). As for HBV, the lowest proportion of HCV positive patients was observed in CC (5.6%).

### *TP53 R249S*-mutated DNA Plasma Concentrations

**Figure 2:**
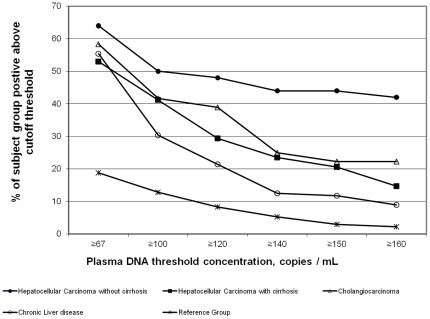
Distribution of the liver disease and reference groups at different cutoffs of positivity of *TP53 R249S* mutation. At 150 copies/mL there is a clear distinction among the three liver cancer groups the chronic liver disease subjects and the reference group. The proportion of patients at this cut-off is higher in HCC/no cirrhosis (44%) than in all other groups (HCC/cirrhosis (21%), CC (22%), CLD (12%) and R (3)).

**Figure 3:**
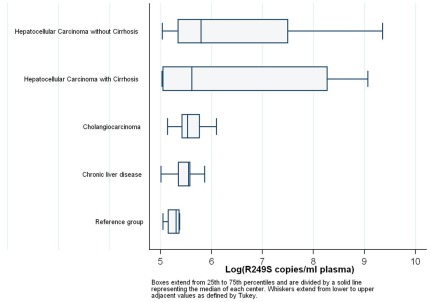
Box and whisker distributions of *TP53 R249S*-*mutated DNA plasma concentrations* (≥150 copies/mL) for the different groups. Boxes extend from 25^th^ to 75^th^ percentiles and are divided by a solid line representing the median of each centre. The median levels for the different groups are: 328 in HCC/no cirrhosis, 273 in HCC/cirrhosis, 252 in CC, 256 in CLD and 202 in R.

Figure 2 shows the proportions of subjects for each study group with detectable *R249S*-mutated DNA at 6 cutoff concentrations from 67 to 160 copies/mL plasma). At the method’s limit of determination (67 copies/mL), the 4 liver disease groups had similar proportions of patients positive for *R249S*-mutated DNA (53–64%), clearly separated from the R group, for which only 19% of subjects were positive. Increasing the cutoff concentration better resolved the 5 study groups. We thus selected a lower cutoff concentration of 150 copies/mL for further comparisons of *R249S*-mutated DNA concentrations between the 4 liver disease groups and the reference group (Figure 3).

Median plasma concentrations of *R249S*-mutated DNA were higher for the HCC/no cirrhosis group (328 copies/mL) than HCC/cirrhosis group (273 copies/mL) with wide concentration ranges for both groups (154–11,576 copies/mL and 152–8,675 copies/mL, respectively). Of note, although the medians for the two HCC groups are similar, the number of individuals in the 70% percentile is higher for HCC/no cirrhosis (n = 11) than for HCC/cirrhosis (n = 3).In contrast, for the CC, CLD and R groups, median plasma concentrations of *R249S*-mutated DNA were lower (252, 256 and 20 copies/mL), respectively, and concentration ranges smaller ([170–446], [150–352] and [155–216]). Taken together, data in Figures 2 and 3 show that (1) high copy numbers of *R249S* were associated with HCC (with or without cirrhosis), whereas CC, CLD and R groups had similarly low *R249S* copy numbers; (2) the proportion of patients with measurable plasma concentrations of *R249S*-mutated DNA was highest in the HCC/no cirrhosis group (44%). Strikingly, this proportion was lower for the HCC/cirrhosis (21%) and CC (22%) groups, which were both higher than CLD (12%) and R (3%) groups. These results indicate that plasma concentrations of *R249S*-mutated DNA occurred most often and at highest concentrations in the HCC/no cirrhosis group, although measurable lower concentrations were observed in the other disease groups.

### Association between Plasma *R249S*-mutated DNA, Chronic Hepatitis Virus Infection and Alpha-fetoprotein Levels

We next examined associations between plasma *R249S*-mutated DNA positivity (>150 copies/mL) and markers of chronic HBV and HCV infections (Table 2) and levels of alpha-fetoprotein (AFP) (Table 3). Table 2 shows that HCC with or without cirrhosis was strongly associated with HBsAg positivity in patients with both high and low plasma concentrations of *R249S*-mutated DNA. However, the strongest association was observed in patients with HCC/no cirrhosis and high plasma concentrations of *R249S*-mutated DNA. In the latter group, the proportion of HBsAg- positive patients (73%) was higher than in patients with HCC/cirrhosis at either high or low plasma concentrations of *R249S*-mutated DNA (43% and 52%, respectively) or patients with HCC/no cirrhosis with low plasma concentrations of *R249S*-mutated DNA (54%). With HCV, a significant association was observed between HCV infection and HCC/cirrhosis, irrespective of plasma concentration of *R249S*-mutated DNA (p = 0.03). Of note, only HCC/cirrhosis was significantly associated with HCV infection when compared to non-HCC subjects (CC, CLD and reference group) (Table 2). Table 3 shows that the distribution of patients with levels of AFP>100 ng/mL was similar between HCC/no cirrhosis and HCC/cirrhosis. However, in the latter group, there was a larger proportion of patients with undetectable or low levels of AFP among HCC/cirrhosis with low plasma concentrations of *R249S*-mutated DNA (56% vs 0%, p = 0.025).

**Table 2:** Relation between Plasma *R249S-*mutated *DNA* and HBs-antigen (HBsAg) and HCV-antibody (HCV-ab).

	Status n (%)				
	Hepatocellular carcinoma without cirrhosis	Hepatocellular carcinoma with cirrhosis	Cholangicarginoma	Chronic liver disease	Reference group
***R249S*** **+**	22 (44)	7 (21)	8 (22)	6 (11)	4 (3)
**HBsAg+**	16 (73) ***	3 (43) *	0	1 (17)	1 (25)
**HBsAg–**	6 (27)	3 (43)	8 (100)	5 (83)	3 (75)
**HBsAg NA**		1 (14)			
***R249S*** **–**	28 (56)	27 (79)	28 (78)	50 (89)	129 (97)
**HBsAg+**	15 (54) ***	14 (52) ***	1 (3.5)	28 (56)	18 (14)
**HBsAg–**	13 (46)	13 (48)	26 (93)	22 (44)	109 (84)
**HBsAg NA**			1 (3.5)		2 (2)
***R249S*** **+**	22 (44)	7 (21)	8 (22)	6 (11)	4 (3)
**HCV+**	3 (14) ^ns^	4 (57) *	1 (13)	1 (1.8)	0
**HCV–**	19 (86)	3 (43)	7 (87)	5 (8.9)	4 (100)
***R249S*** **–**	28 (56)	27 (79)	28 (78)	50 (89)	129 (97)
**HCV+**	4 (14) ^ns^	7 (26) ***	1 (2.8)	12 (24)	0
**HCV–**	24 (86)	20 (74)	27 (75)	38 (76)	129 (100)

*X_2_* test when compared to cholangiocarcinoma, chronic liver disease and reference group all together (*: p value <0.05; ***: p value <0.001; NA: Not Available; ns: non significant).

**Table 3:** Relation between Plasma *R249S-mutated DNA* and AFP.

	Status n(%)				
	Hepatocellular carcinoma without cirrhosis	Hepatocellular carcinoma with cirrhosis	Cholangicarginoma	Chronic liver disease	Reference group
***R249S*** **+**	22 (44)	7 (21)	8 (22)	6 (11)	4 (3)
**AFP<100**	7 (32)	0	7 (87)	6 (100)	4 (100)
**AFP> = 100**	3 (14)	1 (14)	1 (13)	0	0
**AFP> = 400**	12 (54)	6 (86)	0	0	0
***R249S*** ** -**	28 (56)	27 (79)	28 (78)	50 (89)	129 (97)
**AFP<100**	9 (32)	15 (56)	28 (100)	48 (96)	129 (100)
**AFP> = 100**	3 (11)	3 (11)	0	2 (4)	0
**AFP> = 400**	16 (57)	9 (33)	0	0	0
***X_2_*** ** test (** ***p*** ** value)** *	0.950	0.025*			

* *X_2_* test comparing individuals with *R249S* > = 150 copies/mL against individuals with *R249S* <150 copies/mL.

## Discussion

In this study, we have used a highly sensitive and quantitative mass spectrometric method, SOMA, to investigate the relationships between liver cancer and aflatoxin-related plasma *TP53 R249S*-mutated DNA concentrations in free circulating DNA using blood specimens collected in a hospital case: control design. We show that plasma *R249S*-mutated DNA was detected at >67 copies/mL in over 53% of patients with liver cancer or chronic liver disease, and only in 19% of control subjects (including 8% of HBsAg positive subjects). However, when considering only subjects with *R249S*-mutated DNA concentrations *>*150 copies/mL, a clear distinction could be made between the different liver disease statuses. While 44% of HCC/no cirrhosis patients had plasma *R249S*-mutated DNA concentrations greater than 150 copies/mL, this proportion dropped to 21% in patients with HCC/cirrhosis, 22% in CC patients, 12% in CLD patients and 3% in the reference group. This observation indicates that *R249S*-mutated DNA plasma concentrations greater than 150 copies/mL are associated with HCC preferentially developing without cirrhosis. Strikingly, there was no difference in *R249S*-mutated DNA plasma concentrations between HCC developing in a context of cirrhosis and CC, a pathology that originates from non-hepatocyte liver cells and has not been shown to be associated with exposure to aflatoxin. Patients with HCC/no cirrhosis and with high *R249S*-mutated DNA plasma concentrations tended to be more frequently HBsAg positive (73%) than patients with HCC/no cirrhosis with low or undetectable concentrations. Furthermore, in patients with HCC/cirrhosis, the proportion of HBsAg positive subjects was similar, irrespective of *R249S*-mutated DNA plasma concentration. Overall, these results highlight the preferential occurrence of *R249S*-mutated DNA in the plasma of subjects developing HCC in the absence of cirrhosis and/or in subjects who are chronic carriers of HBV.

In a previous study on HCC tissues collected at the NCI, Bangkok, we have detected plasma *R249S*-mutated DNA in 7/26 HCC cases (27%) [15]. Although case: control studies conducted in the early nineties employing biomarkers of aflatoxin exposure have concluded that levels of aflatoxin exposure were low in the Thai population [16], a recent assessment of aflatoxin contamination in two dietary staples in Thailand, peanuts and maize, has reported mean aflatoxin levels which are 3 to 5 times higher than the Thailand regulation limit (20 µg/kg) [23]. Thus levels of dietary exposure to aflatoxin are far from negligible in Thailand and the presence of the mutated DNA in plasma is consistent with the notion that this toxin, together with HBV chronic carriage, remains a major etiological factor for HCC in Thailand. The apparent discrepancy between our detection of plasma *R249S*-mutated DNA and the low levels of aflatoxin biomarkers detected by case: control study in a similar population may be due to the fact that such biomarkers are short-lived in comparison with *TP53 R249S* mutation in liver cells. Thus, in controls, the exposure biomarker is present in only a small proportion of the subjects, whereas in cases, the biomarker is no longer detectable as exposure leading to mutation might have taken place months or years ahead of diagnosis.

In a study on asymptomatic chronic HBV carriers in The Gambia, West Africa, we have found that *R249S*-mutated DNA could be detected at relatively low levels in the plasma of subjects with dietary exposure to aflatoxin, with a seasonal variation that may reflect the dynamics of exposure, mutation formation, elimination of mutated cells and shedding of cell debris into the bloodstream [20]. In the present study, we show that low plasma levels of *R249S-*mutated DNA (>67 copies/mL plasma, compatible with the lower detection limit in asymptomatic subjects from the Gambia) are detectable in a variable proportion of controls, of subjects with chronic liver disease and of liver cancer patients, independently of liver cancer histological type and pathological profile. This low concentration of *R249S-*mutated DNA may occur as the consequence of ongoing exposure to aflatoxin (in controls) and of ongoing destruction of non-cancer cells having acquired a mutation (in patients with CLD, CC, HCC/cirrhosis or HCC/no cirrhosis). However, when only plasma concentrations above a higher cutoff are considered (>150 copies/mL), plasma *R249S*-mutated DNA may essentially, but not exclusively occur as the result of shedding of cellular material by cancer cells containing the mutation. Thus, the high proportion (44%) of patients with HCC/no cirrhosis that are positive above this level may correspond to patients who actually have a liver tumour that contains the mutation. In patients with HCC/cirrhosis or with CC, the proportion of positive subjects above the 150 copies/mL threshold is similar (21 to 22%) but the actual copy numbers are much higher in HCC/cirrhosis patients (up to 8,675 copies/mL) than in CC patients (up to 446 copies/mL) (Figure 3). In patients with CC or CLD, plasma *R249S*-mutated DNA concentrations are actually very low and similar to those observed in the reference group. In the latter group, presence of *R249S* at levels ≥150 copies/mL may correspond to patients with particularly high, ongoing levels of exposure to aflatoxin or who harbor small populations of cells having acquired the mutation. Of the 5 control subjects with plasma *R249S*-mutated DNA concentrations ≥150 copies/mL, only one was positive for HBsAg, indicating that positivity for plasma *R249S*-mutated DNA in control subjects was not restricted to HBV chronic carriers.

In conclusion, the present study provides evidence that acquisition and persistence of the *TP53* mutation *R249S* occurs preferentially in HCC that develops in a context of chronic infection by HBV and without clear clinical evidence of prior or concomitant liver cirrhosis. Although pre-existing cirrhosis is detected in the vast majority (90%) of HCC cases in Western Europe and in the US, it is actually a less frequent phenomenon in HCC occurring in a context of high HBV endemicity/high exposure to aflatoxin although its exact rate of occurrence is poorly documented. In The Gambia, we have observed that cirrhosis was detectable in 62% of patients at the time of HCC diagnosis and this proportion was 59% in patients with plasma *R249S*-mutated DNA [24]. In Thailand, the prospective follow-up of a group of 1810 males HBV carriers aged over 30 over 7 years led to the detection of 36 cases of HCC, 18 (50%) of which developed without any prior evidence of cirrhosis (P.Srivatanakul, unpublished observations). Overall, our results suggest that the development of HCC without pre-existing cirrhosis is enhanced or accelerated in a context of HBV chronic infection and of aflatoxin-induced *TP53 R249S* mutation, suggesting that these two factors cooperate in a distinct pathway of development of hepatocellular carcinoma.
